# Elevated pulse pressure preceded incident chronic kidney disease in the general older population in Sweden

**DOI:** 10.1038/s41598-024-66458-3

**Published:** 2024-07-04

**Authors:** Tomas Månsson, Aldana Rosso, Katarina Ellström, Sölve Elmståhl

**Affiliations:** Division of Geriatric Medicine, Department of Clinical Sciences in Malmö, Lund University and Skåne University Hospital, Jan Waldenströms Gata 35, pl 13, 205 02 Malmö, Sweden

**Keywords:** Nephrology, Risk factors

## Abstract

Arterial stiffness (AS) and chronic kidney disease (CKD) are common in the older population. AS results in increased pulsatile pressure, elevated pulse pressure (PP), and is linked to hypertension. PP is a surrogate for AS. The kidney has low vascular resistance mechanisms, presumably making it vulnerable to the increased pulsatile pressure and hypertension associated with AS. The aims of this study were to investigate the impact of PP elevation on incident CKD (glomerular filtration rate < 60 ml/min/1.73 m^2^) and all-cause mortality. The data was collected from the general population cohort study “Good Aging in Skåne”. Cox proportional hazard regression models adjusted for age, sex, diabetes, and smoking habits were used to investigate the impact of three levels of PP elevation on incident CKD (n = 2693) and all-cause mortality (n = 5253). For PP < 60 mmHg, the median survival time was 18.7 years (event incident CKD) and first quartile survival time (event all-cause mortality) 15.4 years. Elevated PP ≥ 80 mmHg was associated with incident CKD (hazard ratio 1.59, CI 1.28–1.97), but not all-cause mortality. Our results suggest that a finding of PP ≥ 80 mmHg in older age should raise concern of kidney function.

## Introduction

Previously, we have found that chronic kidney disease (CKD), defined as having an estimated glomerular filtration rate (eGFR) < 60 ml/min/1.73 m^2^^1^, is associated with, and also precedes cognitive impairment in a general population of older adults^2,3^. CKD is common in the general older population, and about 75% of subjects above 80 years of age suffer from this condition^4^.

The kidney and the brain are high energy demanding organs, dependent on a stable and continuous blood flow. Compared to other organs, the kidney and the brain have low vascular resistance mechanisms, presumably making both organs vulnerable to pulsatile pressure and hypertension (HT)^5^. Arterial stiffness (AS) results in increased pulsatile pressure and is heavily linked to HT^6^.

Another result of stiffening of the large arteries is increased systolic blood pressure (SBP) and reduced diastolic blood pressure (DBP), that is, elevation of pulse pressure (PP)^7^. Elevation of PP is common in the older population^8^, and elevated PP in the middle-aged and older population has been found to carry an additional risk of cardiovascular events beyond the risk that entails HT alone^9^.

Measurement of carotid-femoral pulse wave velocity (PWV) is considered the gold standard for assessing AS^10^. In more recent years, the use of ultrasound has emerged to assess AS and PWV at a single site through pulse wave imaging, and elastography^11^.

Although carotid-femoral PWV, pulse wave imaging, and elastography can assess AS non-invasively, they still require instruments and additional time that are usually not available to the clinician. Another, more easily assessable and less time-consuming procedure that can be used to assess AS indirectly and non-invasively, is to measure PP. Elevated PP has been linked to cardiovascular disease and death^9,12^, and is commonly used as a surrogate for AS. PP is easily (and quickly) assessed in clinical practice by simply subtracting the DBP from the SBP, taken at rest^10^.

There are a few previous longitudinal studies investigating the impact of AS on incident CKD, as presented in a review on the subject by Garnier et al.^13^. These studies have reported conflicting results, and further studies on the subject are warranted. Elevated PP has been shown to be associated with both cardiovascular mortality and all-cause mortality, as described in a review by Zhao et al.^14^. Most of these studies have not investigated the impact of different levels of elevated PP on mortality.

The aims of this study were to investigate the impact of different levels of elevated PP on incident CKD and/or all-cause mortality in the general older population.

## Methods

### Study population

This study had a longitudinal design. The data was collected from the ongoing cohort study “Good Aging in Skåne” (GÅS). In GÅS, individuals ≥ 60 years of age have randomly been selected for invitation to examination using the population register since 2001. Participants are recruited from both rural and urban areas of the county of Skåne located in the south part of Sweden, representing the general older population. New subjects have been recruited at three-time intervals in the GÅS study. The first group was recruited between 2001 and 2004, the second group was recruited between 2006 and 2012, and the third group between 2012 and 2016. These first visits represented the baseline visit in this study.

Participants in GÅS have been invited to re-examination every 6 years until the age of 78 years, and every 3 years from 78 years of age, until death. Physical and medical examination, neuropsychological testing, anthropometrics, blood sampling, interview, and self-reported questionnaires have been performed at each visit. The GÅS study has been presented in more detail elsewhere^15^. Data for this study was collected from the baseline visits and from every following visit that took place until December 20, 2022.

**Figure 1:**
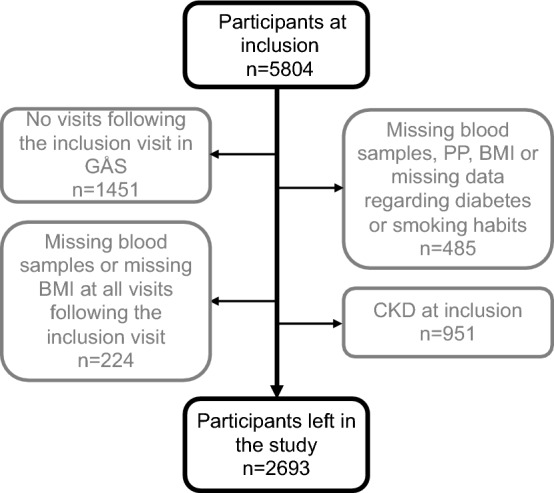
Number of participants (outcome incident CKD). BMI*,* body mass index; CKD*,* chronic kidney disease; GÅS*,* good aging in Skåne; PP, pulse pressure.

The criteria for inclusion and exclusion in the statistical analyses differed depending on the outcome. When CKD was the outcome, the inclusion criteria were absence of CKD (CKD defined as eGFR < 60 ml/min/1.73 m^2^) at the baseline visit, and that the subjects attended at least one visit after the baseline visit. With CKD as outcome, exclusion criteria were missing blood samples, PP, body mass index (BMI), or data regarding diabetes or smoking habits at the baseline visit. A total number of 5804 subjects attended the baseline visit. Of these, 1451 did not attend any further visits to the GÅS study. 485 subjects had missing blood samples, PP, BMI, or data regarding diabetes or smoking habits at the baseline visit. 951 subjects fulfilled the criteria of CKD at the baseline visit. Another 224 subjects had missing blood samples or BMI at all visits following the baseline visit and were therefore excluded. With CKD as outcome, a total of 2693 subjects were included in the calculations, see Fig. 1.

**Figure 2:**
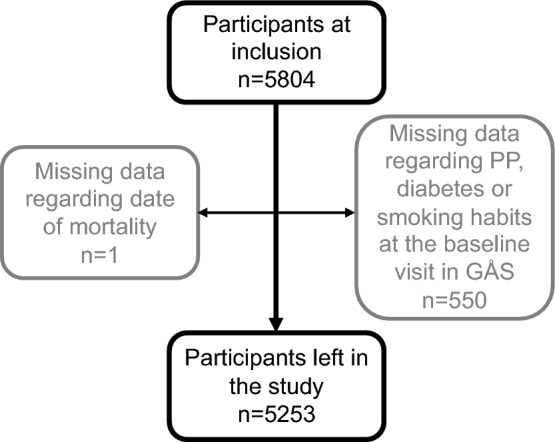
Number of participants (outcome all-cause mortality). CKD*,* chronic kidney disease; GÅS*,* good aging in Skåne; PP*,* pulse pressure.

When the outcome was all-cause mortality, the only inclusion criteria were recorded data regarding age, sex, PP, diabetes, and smoking habits at the baseline visit. Of the 5804 subjects attending the baseline visit, 550 had missing data. One subject had missing data regarding the date of mortality, leaving a total number of 5253 subjects included in the calculations. This is demonstrated in Fig. 2.

### Kidney function

Blood samples were taken non-fasted and cryopreserved to − 80 °C at each examination. The blood samples were later examined at the Skåne University Hospital laboratory in Malmö.

CKD was defined as having an eGFR of < 60 ml/min/1.73 m^2^^1^. Glomerular filtration rate (GFR) was estimated using the reliable Chronic kidney disease epidemiology collaboration (CKD-EPI) equation, in which consideration is taken for age and sex^16^. The updated CKD-EPI equation version from 2012 was used^17^. In non-underweight participants GFR was estimated from both crea and cysC (crea/cysC), since this has been shown to more accurately estimate GFR compared to estimating GFR from either crea or cysC alone^17^. However, crea tends to overestimate GFR in sarcopenic individuals. In the older population sarcopenia is associated with underweight. To avoid underrepresentation of CKD in underweight subjects, GFR was estimated from cysC alone in the group who met the definition of underweight^18^. Underweight was defined as having a BMI of < 23^19^. Weight, measured with light clothes and no shoes, after voiding bowels and bladder in non-fasting conditions, and length were taken by a nurse at each examination^20^.

### Pulse pressure

Blood pressure (BP) was assessed by a physician using a sphygmomanometer. BP was measured in a sitting position in the left arm after a 5-min rest using an appropriately sized cuff (standard 12 cm, smaller 9 cm, or a wider 15 cm). PP was assessed by subtracting the DBP from the SBP. The European Society of Cardiology (ESC) defines having a PP ≥ 60 mmHg as presence of AS in older people^21^. Franklin et al. also found that an elevated PP around this level was associated with future cardiovascular events in the Framingham study^22^. Myers et al.^23^ however, found an association between PP and cardiovascular events first when PP was > 80 mmHg. In line with the ESC, elevated PP was defined as PP ≥ 60 mmHg, and normal PP was defined as < 60 mmHg. Associations between three levels of elevated PP and incident CKD and/or all-cause mortality were examined: 60– < 70 mmHg, 70– < 80 mmHg, and ≥ 80 mmHg.

### Mortality

The date of death was obtained by linkage to the Swedish people and address register from the date of the baseline visit until September 9, 2020. Thus, all individuals were followed up for mortality, regarding additional participation in the GÅS study until emigration or death, whichever occurred first.

### Other covariates

To control for potential confounders masking the hypothetical association between elevated levels of PP and CKD and/or death, the traditional cardiovascular risk factors age, sex, diabetes, and smoking habits were assessed at baseline, and included as covariates in the statistical models^1,9^. HT is heavily linked to AS and PP^6,12^. To avoid collinearity, HT was not included as a covariate.

*Diabetes* was defined as having diabetes mellitus type 1 or 2 at baseline. A history of diabetes was assessed by a physician through interview and by review of the medical records.

*Smoking* was self-reported and defined as either active or former smoker, or never smoked. Smoking status was assessed at baseline.

*BMI* was assessed at baseline. Weight and height were measured by a nurse. The following formula was used to assess BMI: Weight (kg)/Height (m)^2^.

*Cohort* was defined as have been recruited to the GÅS study between 2001 and 2004, between 2006 and 2012, and between 2012 and 2016.

### Statistics

Cox proportional hazard regression models were used to investigate the association between the exposure of three different levels of elevated PP (60– < 70 mmHg, 70– < 80 mmHg, and ≥ 80 mmHg) compared to normal PP (< 60 mmHg) at the baseline visit and the event of incident CKD (eGFR < 60 ml/min/1.73 m^2^) during the follow-up period. Each subject contributed to the analysis from the baseline visit until the event or the last GÅS visit, whichever occurred first. Cox proportional hazard regression models were used to calculate the hazard ratio (HR) for CKD for the different levels of PP elevation compared to normal PP at baseline. The models were first set up unadjusted, and thereafter adjusted for age, sex, diabetes, smoking habits, BMI and cohorts.

In a secondary analysis, the association between elevated PP, set at 60– < 70 mmHg, 70– < 80 mmHg, and ≥ 80 mmHg (< 60 mmHg as reference), at the baseline visit and all-cause mortality was investigated using Cox proportional hazard regression models, both unadjusted and adjusted for the same covariates as above.

To estimate the median and first quartile survival time from the baseline visit until the events, Kaplan–Meier analyzes were used. All three levels of PP elevation mentioned above was examined compared to normal PP, both when the event was incident CKD, and all-cause mortality.

All statistical analyzes were performed using the IBM SPSS software version 29.

### Ethical considerations

The study was approved by the Ethics Committee of Lund University, Sweden (reference number: LU 744–20) and was conducted in accordance with the Declaration of Helsinki. The Division of Geriatric Medicine at Lund University granted and gave permission to access and use the raw data in the study. All subjects or informants gave their written informed consent.

## Results

The baseline characteristics of the participants are shown in Tables 1 and 2.

**Table 1:** Characteristics of the study sample (outcome incident CKD).

Study sample divided by level of PP					
	PP < 60 mmHg	PP 60–≤ 70 mmHg	PP 70–≤ 80 mmHg	PP ≥ 80 mmHg	All
Number	1505	578	309	301	2693
Age in years, mean (SD)	62.9 (5.4)	65.6 (7.6)	67.8 (8.4)	71.0 (9.1)	65.0 (7.3)
Age in years, 25th percentile	60.1	60.3	60.3	60.9	60.2
Age in years, median	60.5	61.0	66.0	67.1	60.8
Age in years, 75th percentile	62.4	66.7	77.7	80.7	66.3
Sex					
Female (%)	740 (49.2)	285 (49.3)	180 (58.3)	178 (59.1)	1383 (51.4)
Male (%)	765 (50.8)	293 (50.7)	129 (41.7)	123 (40.9)	1310 (48.6)
Baseline eGFR^1^ mean (SD)	82.2 (9.6)	80.8 (12.7)	79.6 (11.7)	76.5 (11.7)	81.0 (12.4)
Cardiovascular risk factors					
Hypertension^2^ (%)	615 (40.9)	459 (79.4)	300 (97.1)	301 (100)	1675 (62.2)
SBP^3^ mean (SD)	129.3 (12.4)	145.3 (10.8)	156.7 (11.6)	177.5 (15.4)	141.2 (20.2)
DBP^4^ mean (SD)	81.8 (9.6)	82.0 (10.4)	83.7 (11.7)	86.5 (11.9)	82.6 (10.4)
Diabetes^5^ (%)	64 (4.3)	55 (9.5)	20 (6.5)	23 (7.6)	162 (6.0)
Smoking^6^ (%)	945 (62.8)	371 (64.2)	182 (58.9)	150 (49.8)	1648 (61.2)
BMI^7^ mean (SD)	27.0 (4.3)	27.1 (4.6)	27.2 (4.1)	26.8 (3.7)	27.0 (4.3)
Cardiovascular events					
Coronary insufficiency^8^ (%)	126 (8.4)	55 (9.5)	40 (12.9)	37 (12.3)	258 (9.6)
Stroke^9^ (%)	57 (3.8)	19 (3.3)	17 (5.5)	19 (6.3)	112 (4.2)
Incident CKD (%)	288 (19.1)	165 (28.5)	100 (32.4)	153 (50.8)	706 (26.2)

Study sample divided by CKD incidence					
	Incident CKD	No CKD	All		
Number	706	1987	2693		
Age in years, mean	69.6 (SD 8.5)	63.3 (SD 6.0)	65.0 (7.3)		
Age in years, 25th percentile	60.8	60.1	60.2		
Age in years, median	66.3	60.5	60.8		
Age in years, 75th percentile	78.1	65.9	66.3		
Sex					
Female (%)	401 (56.8)	982 (49.4)	1383 (51.4)		
Male (%)	305 (43.2)	1005 (50.6)	1310 (48.6)		
Baseline eGFR^1^ mean (SD)	72.9 (9.6)	83.9 (12.0)	81.0 (12.4)		
Cardiovascular risk factors					
Hypertension^2^ (%)	512 (72.5)	1163 (58.5)	1675 (62.2)		
SBP^3^ mean (SD)	147.7 (22.3)	139.0 (19.0)	141.2 (20.2)		
DBP^4^ mean (SD)	82.7 (11.2)	82.6 (10.1)	82.6 (10.4)		
Diabetes^5^ (%)	58 (8.2)	104 (5.2)	162 (6.0)		
Smoking^6^ (%)	408 (57.8)	1240 (62.4)	1648 (61.2)		
BMI^7^ mean (SD)	27.2 (4.3)	27.0 (4.3)	27.0 (4.3)		
Cardiovascular events					
Coronary insufficiency^8^ (%)	103 (14.6)	155 (7.8)	258 (9.6)		
Stroke^9^ (%)	39 (5.5)	73 (3.7)	112 (4.2)		

CKD defined as eGFR < 60 ml/min/1.73 m^2^.

^1^eGFR in ml/min/1.73 m^2^.

^2^Hypertension defined as SBP ≥ 140 mmHg and/or DBP ≥ 90 and/or current pharmacological treatment for hypertension. Current pharmacological treatment of hypertension was defined as being treated with any of the following classes of pharmaceuticals with hypertension as indication (Anatomical Therapeutic Chemical codes (ATC) in parenthesis): Antihypertensive agents (ATC02), Diuretics (TC03), Beta-blockers (ATC07), Calcium antagonists (ATC08), RAS inhibitors (ATC09).

^3^SBP in mmHg taken in a sitting position in the left arm after a 5-min rest.

^4^DBP in mmHg taken in a sitting position in the left arm after a 5-min rest.

^5^Diabetes defined as type 1 or type 2 diabetes.

^6^Smoking defined as active or former smoker.

^7^BMI = Weight (kg)/Height (m)^2^.

^8^Coronary insufficiency defined as history of AMI, PTCA, CABG, or angina pectoris.

^9^Stroke defined as history of cerebral infarction, TIA, or RIND.

AHI, acute myocardial ischemia; ATC, anatomical therapeutic chemical codes; BMI, body mass index; CABG, coronary artery bypass graft surgery; CKD, chronic kidney disease; DBP, diastolic blood pressure; eGFR, Estimated glomerular filtration rate; Kg, kilograms; M, meters; PP, pulse pressure; PTCA, percutanous transluminal coronary angioplasty; RIND, reversible ischemic neurological deficit; SBP, systolic blood pressure; SD, standard deviation; TIA, transient ischemic attack.

**Table 2:** Characteristics of the study sample (outcome all-cause mortality).

Study sample divided by level of PP					
	PP < 60 mmHg	PP 60– ≤ 70 mmHg	PP 70– ≤ 80 mmHg	PP ≥ 80 mmHg	All
Number	2535	1083	716	919	5253
Age in years, mean (SD)	65.7 (8.7)	69.8 (10.1)	72.4 (10.3)	76.9 (9.9)	69.4 (10.3)
Age in years, 25th percentile	60.2	60.4	60.7	66.5	60.4
Age in years, median	60.8	66.1	72.0	80.7	66.0
Age in years, 75th percentile	66.4	80.9	81.1	84.0	80.8
Sex					
Female (%)	1280 (50.5)	544 (50.2)	412 (57.5)	595 (64.7)	2831 (53.9)
Male (%)	1255 (49.5)	539 (49.8)	304 (42.5)	324 (35.3)	2422 (46.1)
Cardiovascular risk factors					
Hypertension^1^ (%)	1112 (43.9)	857 (79.1)	692 (96.6)	918 (99.9)	3579 (68.1)
SBP^2^ mean (SD)	128.6 (13.1)	144.4 (11.7)	155.3 (12.2)	175.2 (17.6)	143.6 (22.1)
DBP^3^ mean (SD)	81.0 (10.4)	81.1 (11.3)	82.1 (12.0)	82.8 (12.6)	81.5 (11.2)
Diabetes^4^ (%)	143 (5.6)	111 (10.2)	69 (9.6)	104 (11.3)	427 (8.1)
Smoking^5^ (%)	1586 (62.6)	666 (61.5)	398 (55.6)	421 (45.8)	3071 (58.5)
BMI^6^ mean (SD)	27.1 (4.6)	27.3 (4.6)	27.3 (4.5)	26.8 (4.2)	27.1 (4.5)
Cardiovascular events					
Coronary insufficiency^7^ (%)	323 (12.7)	173 (16.0)	124 (17.3)	202 (22.0)	822 (15.6)
Stroke^8^ (%)	175 (6.9)	79 (7.3)	70 (9.8)	117 (12.7)	441 (8.4)
All-cause mortality	484 (19.1)	330 (30.5)	262 (36.6)	528 (57.5)	1604 (30.5)

Study sample divided by mortality					
	Died during follow-up	Survived during follow-up	All		
Number	1604	3649	5253		
Age in years, mean	78.4 (SD 9.5)	65.4 (SD 7.9)	69.4 (10.3)		
Age in years, 25th percentile	71.9	60.2	60.4		
Age in years, median	81.0	60.8	66.0		
Age in years, 75th percentile	85.5	66.4	80.8		
Sex					
Female (%)	885 (55.2)	1946 (53.3)	2831 (53.9)		
Male (%)	719 (44.8)	1703 (46.7)	2422 (46.1)		
Cardiovascular risk factors					
Hypertension^1^ (%)	1252 (78.1)	2327 (63.8)	3579 (68.1)		
SBP^2^ mean (SD)	151.1 (24.6)	140.4 (20.0)	143.6 (22.1)		
DBP^3^ mean (SD)	80.5 (11.7)	81.9 (11.0)	81.5 (11.2)		
Diabetes^4^ (%)	147 (9.2)	280 (7.7)	427 (8.1)		
Smoking^5^ (%)	826 (51.5)	2245 (61.5)	3071 (58.5)		
BMI^6^ mean (SD)	26.5 (4.5)	27.4 (4.5)	27.1 (4.5)		
Cardiovascular events					
Coronary insufficiency^7^ (%)	400 (24.9)	422 (11.6)	822 (15.6)		
Stroke^8^ (%)	236 (14.7)	205 (5.6)	441 (8.4)		

CKD defined as eGFR < 60 ml/min/1.73 m^2^.

^1^Hypertension defined as SBP ≥ 140 mmHg and/or DBP ≥ 90 and/or current pharmacological treatment for hypertension. Current pharmacological treatment of hypertension was defined as being treated with any of the following classes of pharmaceuticals with hypertension as indication (Anatomical Therapeutic Chemical codes in parenthesis): Antihypertensive agents (ATC02), Diuretics (TC03), Beta-blockers (ATC07), Calcium antagonists (ATC08), RAS inhibitors (ATC09).

^2^SBP in mmHg taken in a sitting position in the left arm after a 5-min rest.

^3^DBP in mmHg taken in a sitting position in the left arm after a 5-min rest.

^4^Diabetes defined as type 1 or type 2 diabetes.

^5^Smoking defined as active or former smoker.

^6^BMI = Weight (kg)/Height (m)^2^.

^7^Coronary insufficiency defined as history of AMI, PTCA, CABG, or angina pectoris.

^8^Stroke defined as history of cerebral infarction, TIA, or RIND.

AHI, acute myocardial ischemia; ATC, Anatomical therapeutic chemical codes; BMI, body mass index; CABG, coronary artery bypass graft surgery; CKD, chronic kidney disease; DBP, diastolic blood pressure; Kg, kilograms; M, meters; PP, pulse pressure; PTCA, Percutanous transluminal coronary angioplasty; RIND, reversible ischemic neurological deficit; SBP, systolic blood pressure; SD, standard deviation; TIA, transient ischemic attack.

### Elevated pulse pressure and incident chronic kidney disease

Participants with elevated PP at the baseline visit more often developed CKD (eGFR < 60 ml/min/1.73 m^2^) during the follow-up period compared to those with normal PP (< 60 mmHg). The CKD incidence increased at higher levels of PP elevation. The CKD incidence during the follow-up period increased from 28.5% for those with PP 60– < 70 mmHg at the baseline visit to 50.8% for those with PP ≥ 80 mmHg. This is demonstrated in Table 1.

In an unadjusted model, all levels of PP elevation at the baseline visit were associated with incident CKD during the follow-up period, and the HR increased at higher levels of PP elevation. Unadjusted, the HR for developing CKD was 1.68 (CI 1.39–2.04, *p*-value < 0.001) for participants with PP 60– < 70 mmHg compared to those with PP < 60 mmHg at baseline. For subjects with PP ≥ 80 mmHg, the HR for incident CKD was 3.37 (CI 2.77–4.10, *p*-value < 0.001). This is demonstrated in Table 3.

**Table 3:** Risk of developing CKD during follow-up according to level of pulse pressure elevation at the baseline visit.

Baseline characteristic	Event	HR	95% CI	*p*-value
Univariate cox proportional hazard regression model (no covariates)				
PP 60–≤ 70 mmHg*	CKD	1.68	1.39–2.04	< 0.001
PP 70–≤ 80 mmHg*	CKD	1.74	1.39–2.19	< 0.001
PP ≥ 80 mmHg*	CKD	3.37	2.77–4.10	< 0.001
Multivariable cox proportional hazard regression model (with covariates)				
PP 60–≤ 70 mmHg*	CKD	1.20	0.99–1.46	0.069
PP 70–≤ 80 mmHg*	CKD	0.98	0.77–1.24	0.865
PP ≥ 80 mmHg*	CKD	1.59	1.29–1.98	< 0.001

CKD defined as eGFR < 60 ml/min/1.73 m^2^.

Statistical method: Univariate and multivariable cox proportional hazard regression models.

Covariates: age, sex, diabetes, smoking, BMI, cohort.

Significance level: 5%

BMI, body mass index; CI, confidence interval; CKD, chronic kidney disease; HR, hazard ratio; PP, pulse pressure.

*PP < 60 mmHg as reference.

After adjustments for covariates, the association between PP ≥ 80 mmHg at baseline and incident CKD during the follow-up period remained. For PP ≥ 80 mmHg, the HR was 1.59 (CI 1.28–1.97, *p*-value < 0.001), as listed in Table 3.

**Figure 3:**
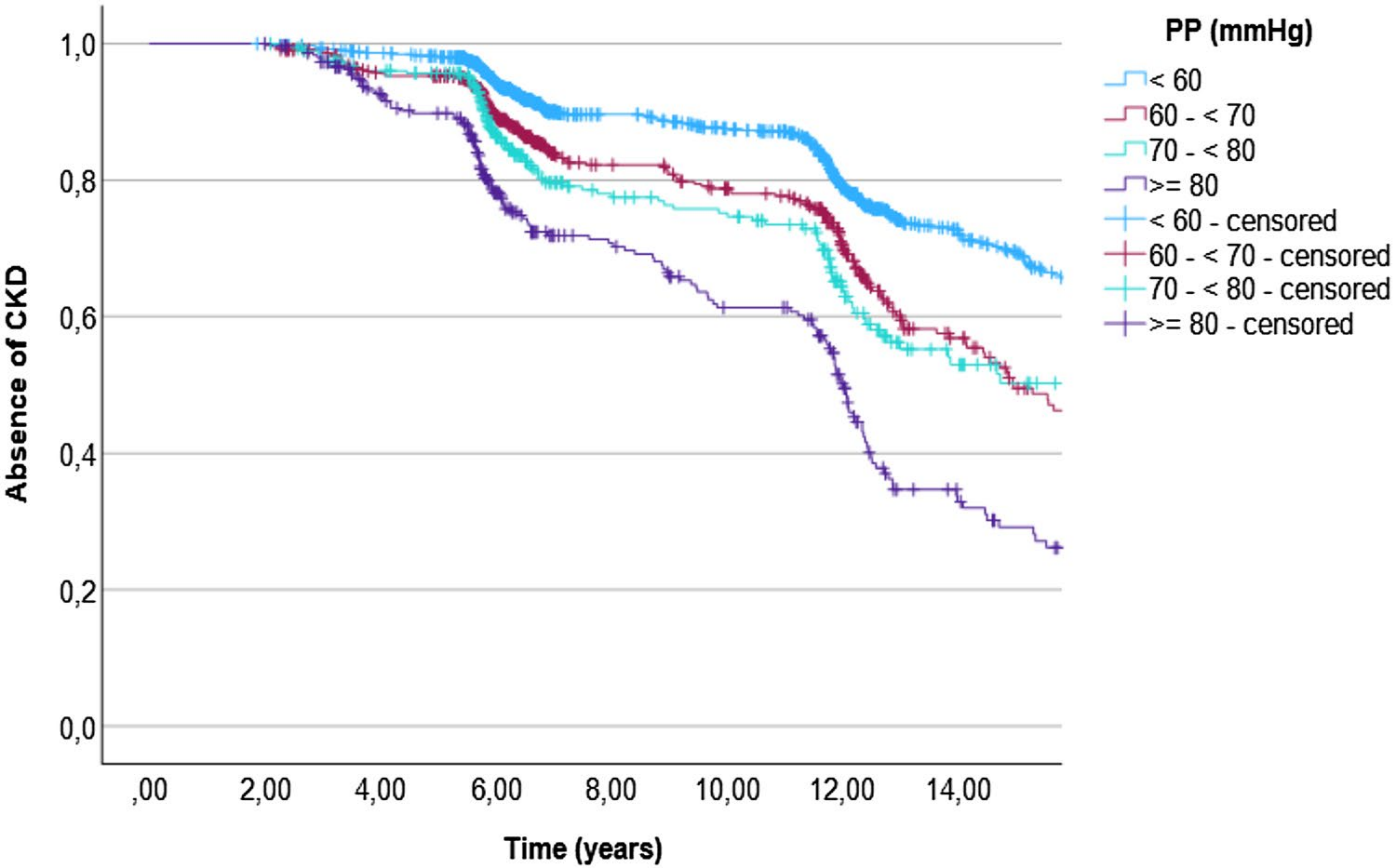
Kaplan–Meier curve for absence of CKD during follow-up. CKD, chronic kidney disease; PP, pulse pressure.

When incident CKD was the event, the Kaplan–Meier estimates of median survival time in years was 15.0 (95% CI 13.8–16.2) for the group with PP 60– < 70 mmHg, 17.4 (95% CI 13.7–21.2) for the group with PP 70– < 80 mmHg, and 12.0 (95% CI 11.7–12.3) for the group with PP ≥ 80 mmHg, compared to 18.7 (95% CI 18.4–19.1) for the group with normal PP (< 60 mmHg). A survival plot is demonstrated in Fig. 3.

### Elevated pulse pressure and all-cause mortality

In an unadjusted model, associations were seen between all levels of PP elevation and all-cause mortality, and the HR increased at higher levels of PP elevation. Unadjusted, the HR for all-cause mortality was 1.63 (CI 1.42–1.88, *p*-value < 0.001) for participants with PP 60– < 70 mmHg (compared to those with PP < 60 mmHg) at the baseline visit. For subjects with PP ≥ 80 mmHg, the HR was 3.50 (CI 3.10–3.96, *p*-value < 0.001). This is demonstrated in Table 4.

**Table 4:** Risk of mortality during follow-up according to level of pulse pressure elevation at the baseline visit.

Baseline characteristic	Event	HR	95% CI	*p*-value
Univariate cox proportional hazard regression model (no covariates)				
PP 60–≤ 70 mmHg*	Mortality	1.63	1.42–1.88	< 0.001
PP 70–≤ 80 mmHg*	Mortality	1.90	1.64–2.21	< 0.001
PP ≥ 80 mmHg*	Mortality	3.50	3.10–3.96	< 0.001
Multivariable cox proportional hazard regression model (with covariates)				
PP 60–≤ 70 mmHg*	Mortality	1.01	0.88–1.17	0.881
PP 70–≤ 80 mmHg*	Mortality	0.88	0.75–1.03	0.112
PP ≥ 80 mmHg*	Mortality	1.05	0.92–1.19	0.516

Statistical method: Univariate and multivariable cox proportional hazard regression models.

Covariates: age, sex, diabetes, smoking, BMI, cohort.

Significance level: 5%

BMI, body mass index; CI, confidence interval; CKD, chronic kidney disease; HR, hazard ratio; PP, pulse pressure.

*PP < 60 mmHg as reference.

After adjustments for covariates, no associations between any level of PP elevation at the baseline visit and all-cause mortality during follow-up remained, as listed in Table 4.

**Figure 4:**
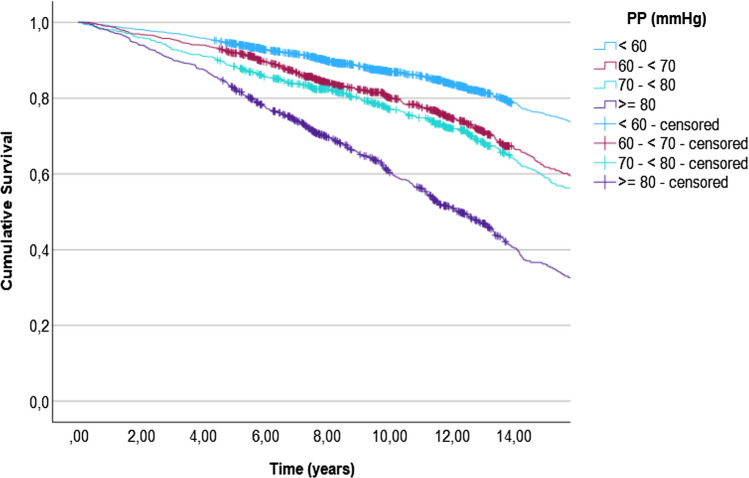
Kaplan–Meier curve for survival during follow-up. PP, pulse pressure.

When all-cause mortality was the event, the median survival time could not be calculated for the reference group (PP < 60 mmHg) and the group with PP 60– < 70 mmHg. Hence, the first quartile survival time was calculated instead. The time in years for survival of the first quartile was 12.0 (95% CI 11.0–12.8) for the group with PP 60– < 70 mmHg, 10.9 (95% CI 9.7–12.3), for the group with PP 70- < 80 mmHg, and 6.8 (95% CI 6.0–7.4) for the group with PP ≥ 80 mmHg, compared to 15.4 (95% CI 14.3–16.2) for the group with normal PP (< 60 mmHg). A survival plot is demonstrated in Fig. 4.

## Discussion

In this study we investigated the impact of PP elevation at three levels (60– < 70 mmHg, 70– < 80 mmHg, and PP ≥ 80 mmHg) on incident CKD and all-cause mortality in a general population cohort of older adults. Elevated PP ≥ 80 mmHg was associated with incident CKD. No level of elevated PP was associated with all-cause mortality. Our findings suggest that PP elevation ≥ 80 mmHg is associated with incident CKD in the general older population.

A sensitivity analysis was made where the statistical model for the impact of elevated PP on incident CKD including the same covariates was re-run with GFR estimated using the CKD-EPI equation based on crea/cysC in all participants. The number of subjects included was 2731. The association remained. The 95% CI for PP 60– < 70 mmHg was 0.98–1.46, *p*-value 0.082. The 95% CI for PP 70– < 80 mmHg was 0.78–1.27, *p*-value 0.963. The 95% CI for PP ≥ 80 mmHg was 1.26–1.96, *p*-value < 0.001.

Analyzes from the Health ABC study (n = 2129), as well as analyzes from the Rotterdam study (n = 3666), showed that AS was associated with incident CKD^24,25^. Analyzes from the Framingham Heart Offspring cohort (n = 1675) however, found no association between AS and incident CKD^26^. The results from these three studies cannot be fully compared to the results of this study. Even though all three studies defined CKD as eGFR < 60 ml/min/1.73 m^2^, they estimated GFR from either cysC or crea, not from crea/cysC, which entails a risk of misclassification regarding CKD status in the non-underweight population. The first two studies did measure PP as a surrogate for AS, but neither one of them investigated the impact of different levels of elevated PP on incident CKD. The last study measured PWV to assess AS, not PP.

Although we did not observe an association between elevated PP and all-cause mortality in this study, it is possible that an association in fact does exist. Most previous longitudinal studies investigating the impact of elevated PP on mortality have indeed found that elevated PP is associated with mortality, as presented in a large review (n = 510,456) on the subject from 2014^14^. The time required to develop CKD is most likely far less than the time required to die. It is possible that the sample size was too small to detect an association between elevated PP and all-cause mortality.

Elevated PP is the result of a large discrepancy between SBP and DBP. However, low DBP itself has been proposed as a risk factor for increased mortality and adverse effects like cardiovascular events, dementia, stroke, and reduced renal function^27–31^. Adverse associations seem to be especially true in the elder population^30^. The increased risk of low DBP can be attributed to reduced organ perfusion but also reversed causation like comorbidities or frailty. In the non-acute setting, low DBP has been associated with AS, excessive anti-hypertensive treatment, heart failure, autonomic dysfunction and hemodynamic properties like low blood volume or exaggerated venous pooling^30,32,33^. To examine if the impact of elevated PP on incident CKD and on all-cause mortality remained independent of DBP, the statistical models were re-run with adjustments made for the same covariates as before but also with DBP included. The results remained essentially the same. The results are presented in Supplementary Tables S1 and S2.

Impairment of the elastic properties of the large arteries due to loss of elastin, fibrosis, and calcification of the arterial vessel walls results in AS, which progresses with age and is very common in the older population^11^. HT is strongly associated with AS^6^. If HT is a cause of AS or vice versa is debated^34^. AS causes reduced compliance in the large arteries. This results in elevated PWV and increased pulsatile pressure, which is insufficiently dampened by the resistance arteries, leading to increased stress to the microcirculation of end organs^6^. The kidney and the brain are high energy consuming organs and depend on a stable and continuous blood flow. The microvasculature of the kidney and the brain are likely highly sensitive to the increased PWV and pulsatile pressure accompanying AS, since both the kidney and the brain have low vascular resistance properties compared to other organs^5^.

CKD is heavily linked to cardiovascular disease, but if CKD is a result of vascular damage or vice versa (or both) is less known^35^. The kidney is likely sensitive to vascular damage for reasons described above. Since this was an observational study, even though AS preceded CKD, it is difficult to make any causal assumptions. A theory is that AS could be a contributing cause of CKD, at least initially in the pathological process. CKD can most likely in turn induce vascular damage through increased inflammation and uremic toxins^36^, and as CKD and vascular damage worsen, cause and effect are likely to become increasingly difficult to establish.

Elevated PP is a surrogate for AS^10^. PP can be assessed easily by simply subtracting the DBP from the SBP measured in rest. The only instruments needed are a blood pressure cuff and a stethoscope, which are usually included in the standard equipment of a standard clinical practice. In this study, PP ≥ 80 mmHg was associated with incident CKD in the general older population. PP elevation is common in the older population^8^, and has previously been shown to increase the risk of cardiovascular events beyond the risk posed by HT alone^9^. A finding of PP ≥ 80 mmHg should raise concern regarding kidney function and possibly the cardiovascular status of the older patient. We recommend assessment of kidney function through estimation of GFR in case of a finding of PP ≥ 80 mmHg. In case of a finding of PP ≥ 80 mmHg, we also suggest that evaluation and management of traditional, treatable cardiovascular risk factors, such as hypertension, diabetes, and smoking habits are considered.

A strength of this study includes the large number of participants representing the general older population, as well as the long follow-up period. Another strength is that GFR was estimated from both crea and cysC in the non-underweight, and that consideration was taken to possible misclassification in the underweight by only estimating GFR from cysC in this group. Furthermore, using the established cut-off limit of eGFR < 60 ml/min/1.73 m^2^ to define CKD enables to study mechanisms of early signs of CKD in the general older population. Another strength was the use of three levels of PP elevation.

This study had some limitations. Since almost all subjects with elevated PP (≥ 60 mmHg) also had HT (89.2%), no stratification could be made based upon HT status. Therefore, this study could not determine if elevated PP is associated with incident CKD or all-cause mortality independent of HT. Since we had no data regarding the cause of death of the subjects who died during follow-up, we could not determine if PP elevation was associated with cardiovascular mortality. Microalbuminuria was not measured during the study, which may have resulted in underestimation of true association due to a dilution effect, as the eGFR limit < 60 ml/min/1.73 m^2^ may be part of normal ageing. The invitation interval was longer for participants who were younger than 78 years of age. Therefore, changes in their kidney function were detected every 6 years. By study design, any decline in kidney function before that time is noted later for younger participants. Chronic kidney disease is a complex disease with several risk factors, such as age, sex, diabetes, hypertension, obesity, environmental factors, etc^37^. Despite the inclusion of the main risk factors in the statistical models, residual confounding remains and therefore the results presented in this work should be interpreted with caution. According to protocol, participants with SBP ≥ 140 mmHg and/or DBP ≥ 90 at the baseline visits were recommended follow-up for hypertension at their general practitioner’s office. As this procedure constitutes an intervention, it is possible that the observed events of PP elevation were underrepresented. Even though home visits were offered to those too frail to attend the GÅS study central, it is possible that the most frail individuals were underrepresented in this study. Even though PP is an established surrogate for AS, it is dependent of other factors than AS, such as aortic geometry and peak aortic flow, and therefore probably not as precise as measuring carotid-femoral PWV to assess AS^10^. However, since measurement of carotid-femoral PWV to assess AS requires special equipment and is time consuming, carotid-femoral PWV is probably most usable in research, whereas the use of PP as a surrogate for AS is more suitable in the clinical situation due to its accessibility.

In conclusion, the authors suggest that a finding of PP ≥ 80 mmHg in an older subject should raise concern of kidney function and renewed assessment of kidney function by estimating GFR. The authors also believe that the finding of PP ≥ 80 mmHg should raise concern of vascular status, and that evaluation and management of treatable cardiovascular risk factors, such as hypertension, diabetes, and smoking habits, are considered.

### Supplementary Information

Supplementary Table S1.

Supplementary Table S2.

## Data Availability

The data was based on the GÅS dataset. The GÅS dataset is restricted and not publicly available. The data can be obtained by the co-author and GÅS research leader professor Sölve Elmståhl by reasonable request.
