# Supplementary material for: Elevated pulse pressure preceded incident chronic kidney disease in the general older population in Sweden
Source: Sci Rep. 2024 Jul 4;14:15414. doi: 10.1038/s41598-024-66458-3 (PMC11224232; doi:10.1038/s41598-024-66458-3)
Supplement: Supplementary file 1 — Supplementary Table S1. [file 41598_2024_66458_MOESM1_ESM.docx]

| **Supplementary Table S1**. Risk of developing CKD during follow-up according to level of pulse pressure elevation at the baseline visit | | | | |
| --- | --- | --- | --- | --- |
| Univariate cox proportional hazard regression model (no covariates) | | | | |
| Baseline characteristic | Event | HR | 95 % CI | p-value |
| PP 60 - ≤ 70 mmHg* | CKD | 1.68 | 1.39-2.04 | <0.001 |
| PP 70 - ≤ 80 mmHg* | CKD | 1.74 | 1.39-2.19 | <0.001 |
| PP ≥ 80 mmHg* | CKD | 3.37 | 2.77-4.10 | <0.001 |
| Multivariable cox proportional hazard regression model (with covariates) | | | | |
| Baseline characteristic | Event | HR | 95 % CI | p-value |
| PP 60 - ≤ 70 mmHg* | CKD | 1.18 | 0.97-1.45 | 0.100 |
| PP 70 - ≤ 80 mmHg* | CKD | 0.98 | 0.77-1.25 | 0.882 |
| PP ≥ 80 mmHg* | CKD | 1.47 | 1.17-1.85 | <0.001 |
| *PP < 60 mmHg as reference | |  |  |  |
| CKD defined as eGFR < 60 ml/min/1.73m^2^ | |  |  |  |
| Statistical method: Univariate and multivariable cox proportional hazard regression models | | | | |
| Covariates: age, sex, diabetes, smoking, BMI, DBP, cohort | |  |  |  |
| Significance level: 5 % |  |  |  |  |
| Abbreviations: BMI = Body mass index, CI = Confidence interval, CKD = Chronic kidney disease, DBP = Diastolic blood pressure, HR = Hazard ratio, PP = pulse pressure | | | | |
