# Supplementary material for: Elevated pulse pressure preceded incident chronic kidney disease in the general older population in Sweden
Source: Sci Rep. 2024 Jul 4;14:15414. doi: 10.1038/s41598-024-66458-3 (PMC11224232; doi:10.1038/s41598-024-66458-3)
Supplement: Supplementary file 2 — Supplementary Table S2. [file 41598_2024_66458_MOESM2_ESM.docx]

| **Supplementary Table S2**. Risk of mortality during follow-up according to level of pulse pressure elevation at the baseline visit | | | | |
| --- | --- | --- | --- | --- |
| Univariate cox proportional hazard regression model (no covariates) | | | | |
| Baseline characteristic | Event | HR | 95 % CI | p-value |
| PP 60 - ≤ 70 mmHg* | Mortality | 1.63 | 1.42-1.88 | <0.001 |
| PP 70 - ≤ 80 mmHg* | Mortality | 1.90 | 1.64-2.21 | <0.001 |
| PP ≥ 80 mmHg* | Mortality | 3.50 | 3.10-3.96 | <0.001 |
| Multivariable cox proportional hazard regression model (with covariates) | | | | |
| Baseline characteristic | Event | HR | 95 % CI | p-value |
| PP 60 - ≤ 70 mmHg* | Mortality | 1.02 | 0.89-1.18 | 0.755 |
| PP 70 - ≤ 80 mmHg* | Mortality | 0.91 | 0.77-1.06 | 0.215 |
| PP ≥ 80 mmHg* | Mortality | 1.08 | 0.94-1.24 | 0.271 |
| *PP < 60 mmHg as reference |  |  |  |  |
| Statistical method: Univariate and multivariable cox proportional hazard regression models | | | | |
| Covariates: age, sex, diabetes, smoking, BMI, DBP, cohort | |  |  |  |
| Significance level: 5 % |  |  |  |  |
| Abbreviations: BMI = Body mass index, CI = Confidence interval, CKD = Chronic kidney disease, DBP = Diastolic blood pressure, HR = Hazard ratio, PP = pulse pressure | | | | |
